# Plasma proteomic profiling uncovers the age-related molecular changes in chronic obstructive pulmonary disease

**DOI:** 10.1186/s12890-026-04396-8

**Published:** 2026-06-10

**Authors:** Yanpeng Li, Li Yang, Chengshui Chen, Zhangli Hu, Jian Zeng, Yun Wang, Shan Zhong

**Affiliations:** 1 School of Basic Medical Sciences, Shenzhen University Medical School, No. 1066 Xueyuan Ave, Nanshan District, Shenzhen, Guangdong 518055 People’s Republic of China; 2 Department of Respiratory Medicine, The First Affiliated Hospital of Wenzhou Medical University, Wenzhou, Zhejiang 325000 People’s Republic of China; 3 Guangdong Provincial Key Laboratory of Functional Substances in Medicinal Resources and Healthcare Products, School of Life Sciences and Food Engineering, Hanshan Normal University, Chaozhou, Guangdong 521041 People’s Republic of China; 4 Longhua Innovation Institute for Biotechnology, College of Life Sciences and Oceanography, Shenzhen University, No. 1066 Xueyuan Ave, Nanshan District, Shenzhen, Guangdong 518055 People’s Republic of China; 5 Institute of Precision Medicine, Peking University Shenzhen Hospital, Shenzhen, Guangdong 518036 People’s Republic of China

**Keywords:** COPD, Aging, Proteome, Plasma, Biomarker

## Abstract

**Background:**

Chronic obstructive pulmonary disease (COPD) is a leading cause of morbidity and mortality worldwide, with aging as a major risk factor. This study aimed to investigate age-related proteins potentially involved in COPD pathogenesis.

**Methods:**

A total of 70 plasma samples (35 healthy controls, 35 COPD patients) were analyzed by data-independent acquisition mass spectrometry (DIA-MS), with each group stratified into young (≤ 50 years) and aged (≥ 70 years) subgroups. Differentially expressed proteins were identified using the limma package in R, followed by functional enrichment and protein–protein interaction analyses. Spearman’s correlation and multivariable linear regression were used to assess associations between protein levels and lung function. Receiver operating characteristic curves (ROC) were generated to evaluate the diagnostic value of aging-related proteins in COPD.

**Results:**

A total of 534 proteins were detected by DIA-MS. Following missing value imputation and quality filtering, 413 proteins were retained for analysis. Comparative analysis revealed 20 proteins differentially expressed between young and aged COPD patients (11 upregulated, 9 downregulated), which were significantly enriched in pathways including glutathione metabolism, nitrogen metabolism, endoplasmic reticulum protein processing, and PI3K-Akt signaling. Notably, expression levels of polymeric immunoglobulin receptor (PIGR), thrombospondin-1 (TSP1), secreted protein acidic and rich in cysteine (SPRC), and adiponectin (ADIPO) were correlated with FEV₁/FVC ratio in COPD patients; PIGR and ADIPO remained independently associated after adjusting for covariates. An exploratory model integrating these four proteins with age demonstrated reasonable discriminatory ability for COPD (AUC 0.818; 95% CI: 0.718–0.917).

**Conclusion:**

Our findings identify four plasma proteins—PIGR, SPRC, TSP1, and ADIPO—as age-related biomarkers in COPD that may offer value for patient stratification and endotyping. These results provide new insights into the role of aging in COPD pathogenesis and warrant further validation.

**Supplementary Information:**

The online version contains supplementary material available at 10.1186/s12890-026-04396-8.

## Introduction

Chronic Obstructive Pulmonary Disease (COPD), a clinically heterogeneous respiratory disorder characterized by chronic respiratory symptoms resulting from inflammation of the small airways and/or alveoli, leads to persistent and progressive airflow obstruction. As a major contributor to the global burden of disease, COPD significantly impacts mortality and disability worldwide [1, 2]. In China, the prevalence of COPD among individuals over 40 years old has risen to 13.7%, affecting approximately 100 million people [3]. While tobacco smoking remains a primary risk factor for COPD development, other contributing factors are increasingly recognized. Epidemiological studies reveal that the elderly population exhibits 2–3 times higher COPD prevalence globally, with China demonstrating an even more pronounced disparity of over 3.7-fold compared to younger populations [3, 4], this pattern strongly suggests aging as a critical risk factor in COPD pathogenesis [5, 6]. With increasing global life expectancy, age-related diseases like COPD are projected to escalate further, imposing substantial strain on healthcare infrastructures and socioeconomic systems [7]. Consequently, elucidating the mechanistic interplay between aging processes and COPD progression becomes imperative for developing effective intervention strategies.

Aging, characterized by 12 interconnected hallmarks, represents a progressive decline in homeostatic maintenance that predisposes organisms to chronic diseases and mortality [8]. Notably, multiple hallmarks of aging—particularly cellular senescence, chronic inflammation, epigenetic dysregulation, and proteostasis collapse—demonstrate mechanistic convergence with COPD progression [6, 9]. Emerging research delineates the critical involvement of conserved aging-related pathways in COPD pathogenesis, including the Sirtuin-mTOR axis and FGF23/Klotho signaling network [10]. Clinical investigations reveal significant downregulation of anti-aging mediators (Sirtuin1 and Klotho) coupled with elevated expression of senescence marker *P21* in COPD patients compared to healthy controls, suggesting their potential utility as diagnostic biomarkers [5]. Therapeutic exploration demonstrates that geroprotectors such as resveratrol and melatonin ameliorate COPD pathology through dual mechanisms: suppression of pro-inflammatory cytokine cascades and restoration of Sirtuin1 expression [11, 12]. Despite established epidemiological associations between aging and COPD predominance, the molecular underpinnings of this relationship remain poorly delineated. Additional investigations are warranted to decipher aging-associated pathomechanisms in COPD and identify more effective targets for anti-aging therapies.

Proteomic investigations integrated within multi-omics frameworks have unveiled novel mechanistic insights into COPD pathogenesis [13, 14]. The evolution of mass spectrometry (MS)-based proteomics over recent decades has revolutionized biomarker discovery, enabling precise therapeutic targeting through high-resolution protein profiling. Proteomic analyses of blood and sputum specimens have identified several prognostic biomarkers, including C-reactive protein (CRP), fibrinogen, fetuin-B (FETUB), surfactant protein D (SP-D), neutrophil elastase (NE), matrix metalloproteinases (MMPs), and immunoglobulins [15–17]. These findings demonstrate the value of proteomic approaches in delineating protein-centric pathomechanisms underlying COPD progression. Notably, the application of proteomics to elucidate aging-COPD interrelationships remains scarce.

**Fig. 1:**
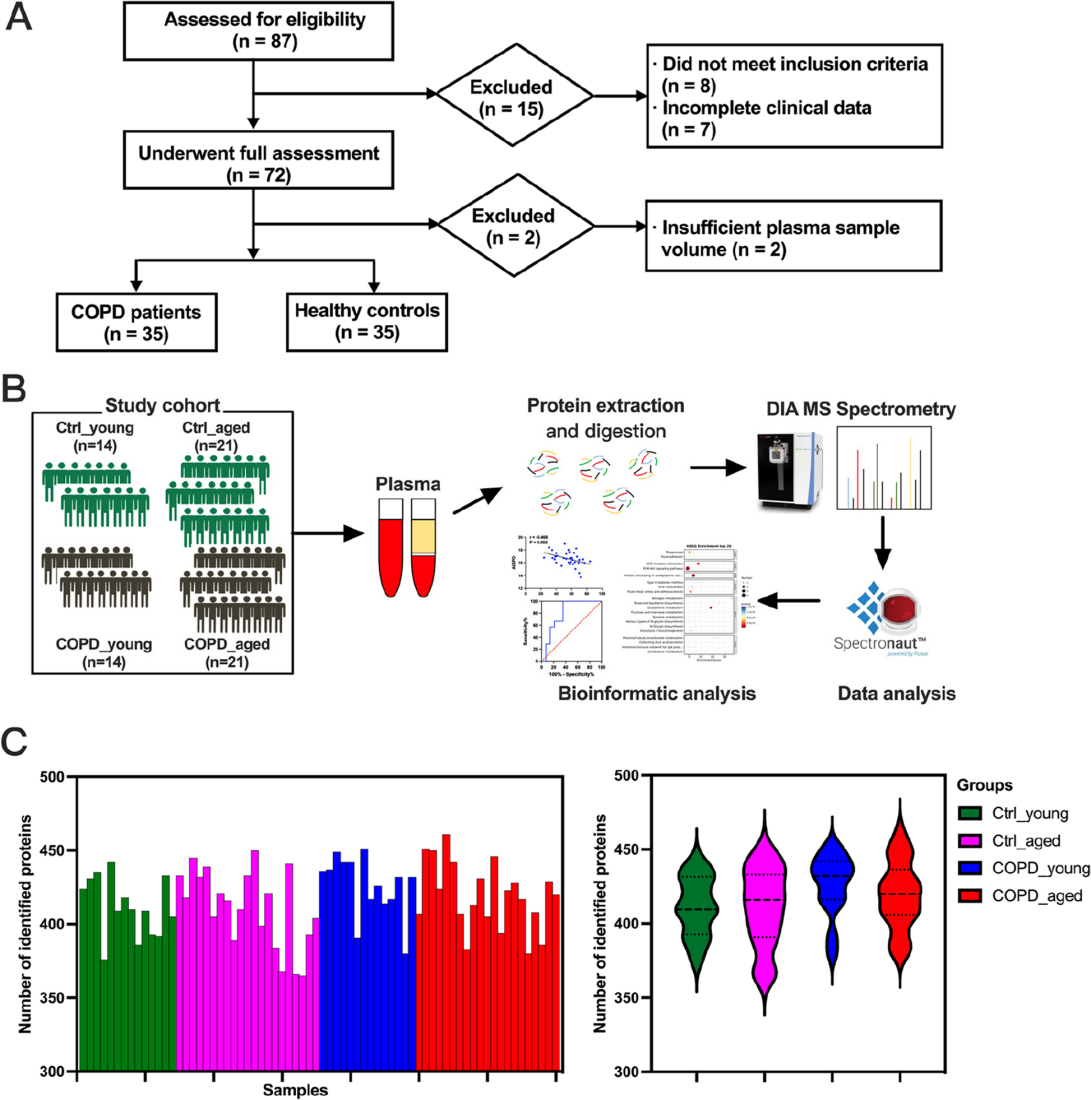
Experimental design and plasma proteome profiling results. **A** CONSORT flow diagram of participant enrollment and subgrouping. **B** Schematic of the DIA-MS-based proteomic workflow. **C** Distribution of identified proteins across the four experimental groups

In this study, we aim to employ quantitative proteomic profiling of clinical plasma specimens (Fig. 1A and B) to systematically investigate aging-associated molecular perturbations in COPD pathophysiology, which will advance our mechanistic understanding of the aging-COPD axis, ultimately informing precision medicine approaches for elderly patients with COPD.

## Material and methods

### Clinical subjects

This study was approved by the Ethics Committee of the First Affiliated Hospital of Wenzhou Medical University (Approval No. 2016131). All participants provided written informed consent prior to enrollment. The study was conducted in accordance with the Declaration of Helsinki. A total of 87 potential participants were initially screened. Fifteen individuals were excluded: eight did not meet the inclusion criteria, and seven had incomplete clinical data. The remaining 72 participants underwent full assessment, of whom two were subsequently excluded due to insufficient plasma sample volume. Thus, 70 participants were included in the final proteomic analysis (As shown in Fig. 1A). No samples were excluded after proteomic data acquisition.

The 70 participants comprised 35 COPD patients and 35 age-matched healthy controls. Young subjects were defined as those aged 50 years or younger (Ctrl_young, n = 14; COPD_young, n = 14), while aged subjects were defined as those aged 70 years or older (Ctrl_aged, n = 21; COPD_aged, n = 21) [18]. Inclusion and exclusion criteria followed established protocols [19]. Briefly, COPD diagnosis was based on the Global Initiative for Chronic Obstructive Lung Disease (GOLD) criteria, requiring respiratory symptoms and a post-bronchodilator forced expiratory volume in 1 s (FEV_1_)/forced vital capacity (FVC) ratio < 70%. Patients with other obstructive lung diseases (e.g., asthma) or severe/active lung infections were excluded.

### Sample preparation for LC–MS/MS analysis

For protein extraction, 50 μL of lysis buffer (8 M urea in 100 mM tetraethylammonium bromide, TEAB) was added to 5 μL of plasma. The mixture was incubated at 32 °C for 30 min. Proteins were then reduced with 10 mM Tris(2-carboxyethyl)phosphine (TCEP) at 32 °C for 30 min, followed by alkylation with 200 mM iodoacetamide (IAA) for 30 min at room temperature in the dark. The samples were diluted with four volumes of 100 mM TEAB. Protein concentration was quantified using a bicinchoninic acid (BCA) assay kit (Sangon Biotech, China) following the manufacturer’s protocol. After quantification, trypsin (Promega, USA) was added at a 1:50 (w/w) trypsin-to-protein ratio for overnight digestion at 37 °C. The digested peptides were desalted using an Oasis HLB 1 cc Vac Cartridge column (Waters, USA) per the manufacturer’s instructions, eluted with 50% acetonitrile/0.1% trifluoroacetic acid aqueous solution, and vacuum-dried at room temperature. Samples were stored at −80 °C until further use.

### DIA-LC–MS/MS analysis

Peptides were reconstituted in 50 μL of 0.1% aqueous formic acid. For LC–MS/MS analysis, 1 μg of peptides per sample was loaded onto an ultra-performance liquid chromatography (UPLC) system (Ultimate 3000, Thermo Fisher Scientific, USA) coupled to an Orbitrap Exploris 480 mass spectrometer equipped with a nano-electrospray ion source. Mobile phase A consisted of 0.1% formic acid in water (99.9% water/0.1% formic acid, v/v), and mobile phase B was 80% acetonitrile/0.1% formic acid in water (80% acetonitrile/20% water, v/v). Peptide separation was performed on an Acclaim™ PepMap™ RSLC analytical column (75 μm inner diameter × 25 cm length, 2 μm particle size; Thermo Fisher Scientific, USA) at 50 °C with a constant flow rate of 300 nL/min. The gradient program was as follows: 3% A (0–3 min), 8% A (3–86 min), 35% A (86–96 min), 40% A (96–100 min), and 90% A (100–110 min).

Data-independent acquisition (DIA) was performed in positive ion mode. Full MS scans covered a mass-to-charge (m/z) range of 300–1200 with a resolution of 120,000. For MS/MS, a stepped collision energy (25%, 30%, 35%) was applied, with fragment spectra acquired at a resolution of 30,000 (at m/z 120).

### Mass spectrometry raw data analysis

All samples were processed and analyzed in a single batch to minimize technical variability. Therefore, no batch correction was applied. DIA raw data were processed and analyzed using Spectronaut Pulsar software (version17, Biognosys, USA). The software was configured to search against the UniProt_HomoSapiens_20367 database, with a false discovery rate (FDR) cutoff of ≤ 1% at both precursor and peptide levels. Remaining parameters were maintained at default settings.

### Differentially expressed proteins identification

Prior to differential expression analysis, proteins were filtered using the 50% rule: only proteins with valid intensity values in at least 50% of samples were retained for further analysis. This resulted in 413 proteins from the initial 534 detected. Missing values in the filtered dataset were then imputed using the k-nearest neighbors (KNN) algorithm with k = 5. The imputed data were log2-transformed to achieve approximately normal distribution before input into limma. The limma linear model included group assignment (Ctrl_young, Ctrl_aged, COPD_young, COPD_aged) as the primary variable of interest. To account for multiple testing, p-values from thelimma analysis were adiusted using the Benjamini-Hochbergprocedure to control the false discovery rate (FDR) at 5%. Proteins with *P* < 0.05 were considered statistically significant. To prioritize biologically meaningful changes, only those with a |log₂(fold change)|> 0.585 were retained for further analysis.

### Functional and pathway enrichment analysis

Functional annotations of critical aging-associated proteins in COPD were performed using the Omicshare online platform (https://www.omicshare.com/). Gene Ontology (GO) enrichment analysis was conducted to evaluate potential biological roles across three categories: molecular function, cellular component, and biological process. Additionally, Kyoto Encyclopedia of Genes and Genomes (KEGG) pathway enrichment analysis was employed to identify biologically relevant signaling pathways.

### Protein–protein interaction (PPI) network analysis

Protein–protein interaction analysis was performed using the Search Tool for the Retrieval of Interacting Genes/Proteins (STRING) database (version 12.0; https://cn.string-db.org/).

### Correlation and regression analysis

To evaluate the association between plasma protein levels and lung function (FEV₁/FVC ratio), Spearman's rank correlation coefficients were calculated for each of the differentially expressed proteins. Multivariable linear regression models were then fitted with FEV₁/FVC as the dependent variable and candidate protein as the independent variable, adjusting for sex, smoking status (never, former, current), and body mass index (BMI). Results are presented as unstandardized regression coefficients (β) with 95% confidence intervals (CIs).

### Receiver operating characteristic (ROC) curve analysis

To evaluate the potential clinical utility of the identified age-related proteins, we constructed a multivariable logistic regression model to predict COPD status (yes/no). The model included the key proteins and age as continuous variables. Model performance was assessed using ROC curve analysis with tenfold cross-validation to estimate out-of-sample performance. The area under the curve (AUC) with 95% confidence interval was calculated.

### Statistical analysis

Statistical analyses were performed using R software (version 4.2.3) and GraphPad Prism 9 (GraphPad Software, USA). For two-group comparisons, data were analyzed using Student’s *t*-test (parametric) or the Mann–Whitney U-test (non-parametric), depending on normality. Results are expressed as mean ± standard deviation (SD). A two-sided *P* < 0.05 was considered statistically significant.

## Results

### Subjects’ characteristics

This study enrolled 70 participants in a case–control design, comprising 35 COPD patients and 35 age-matched healthy controls. The clinical characteristics of these subjects are listed in Table 1. While gender distribution and smoking status showed no significant intergroup differences, COPD patients demonstrated significantly impaired pulmonary function (FEV_1_/FVC%: 54.492 ± 11.724 vs 83.963 ± 8.050,* P* < 0.0001; FEV_1_% predicted: 55.869 ± 22.606 vs 88.000 ± 15.686, *P* < 0.0001) and lower BMI (22.160 ± 2.984 kg/m^2^ vs 23.709 ± 2.759 kg/m^2^, *P* = 0.0273). The elderly subgroup (≥ 70 years old) exhibited accelerated pulmonary decline in COPD, with significantly reduced FEV1/FVC% (50.281 ± 11.794 vs 60.807 ± 8.597, *P* = 0.0072) and FEV_1_% predicted (46.590 ± 15.361 vs 69.786 ± 25.017,* P* = 0.0017) compared to younger counterparts (≤ 50 years old).

**Table 1:** Basic characteristics of COPD and control subjects

	**Control**			**COPD**		
	**Total** **(*n* = 35)**	**Ctrl_young (*n* = 14)**	**Ctrl_aged (*n* = 21)**	**Total** **(*n* = 35)**	**COPD_young** **(*n* = 14)**	**COPD_aged** **(*n* = 21)**
Male/Female	30/5	13/1	17/4	33/2	13/1	20/1
Age, Mean ± SD	61.571 ± 14.086 ^a1^	44.929 ± 2.759 ^b^	72.667 ± 3.039	62.971 ± 14.985	45.214 ± 2.887 ^c1^	74.810 ± 2.909
BMI, Mean ± SD	23.709 ± 2.759	24.168 ± 2.855	23.403 ± 2.719	22.160 ± 2.984	22.340 ± 3.052	22.040 ± 3.008
Current/ex-smokers	8/26	4/10	4/16	15/29	6/13	9/16
Pulmonary functions, Mean ± SD						
FEV1/FVC %	83.963 ± 8.050 ^a2^	85.946 ± 6.762	82.641 ± 8.709	54.492 ± 11.724	60.807 ± 8.597 ^c2^	50.281 ± 11.794
FEV1% predicted	88.000 ± 15.686 ^a3^	96.193 ± 11.321	82.538 ± 16.020	55.869 ± 22.606	69.786 ± 25.017 ^c3^	46.590 ± 15.361

^a1^ *P* = 0.0273, ^a2^ *P* < 0.0001, ^a3^ *P* < 0.0001 indicates between total Control and COPD

^b^ *P* < 0.0001 indicates between Ctrl_young and Ctrl_aged

^c1^ *P* < 0.0001, ^c2^ *P* = 0.0072, ^c3^ *P* = 0.0017 indicates between COPD_young and COPD_aged

### Proteomic characteristics of all plasma subjects

Plasma proteomic profiling was performed on all 70 clinical specimens using data-independent acquisition mass spectrometry (DIA-MS), yielding 534 unique protein groups (Supplementary File 1). Of these, 252 proteins were detectable across all 70 samples. The average numbers of proteins identified in the Ctrl_young, Ctrl_aged, COPD_young, and COPD_aged groups were 411, 411, 426, and 419, respectively (Fig. 1C), with no significant differences in either the quantity or compositional profiles of detected proteins across the four subgroups. Following application of a 50% missing value threshold and K-nearest neighbor (KNN) imputation, 413 high‑quality proteins were retained for downstream analysis. The protein-level missing rate—defined as the proportion of proteins excluded due to excessive missingness—was approximately 22.7% (121/534).

### Identification of aging-associated DEPs in COPD

**Fig. 2:**
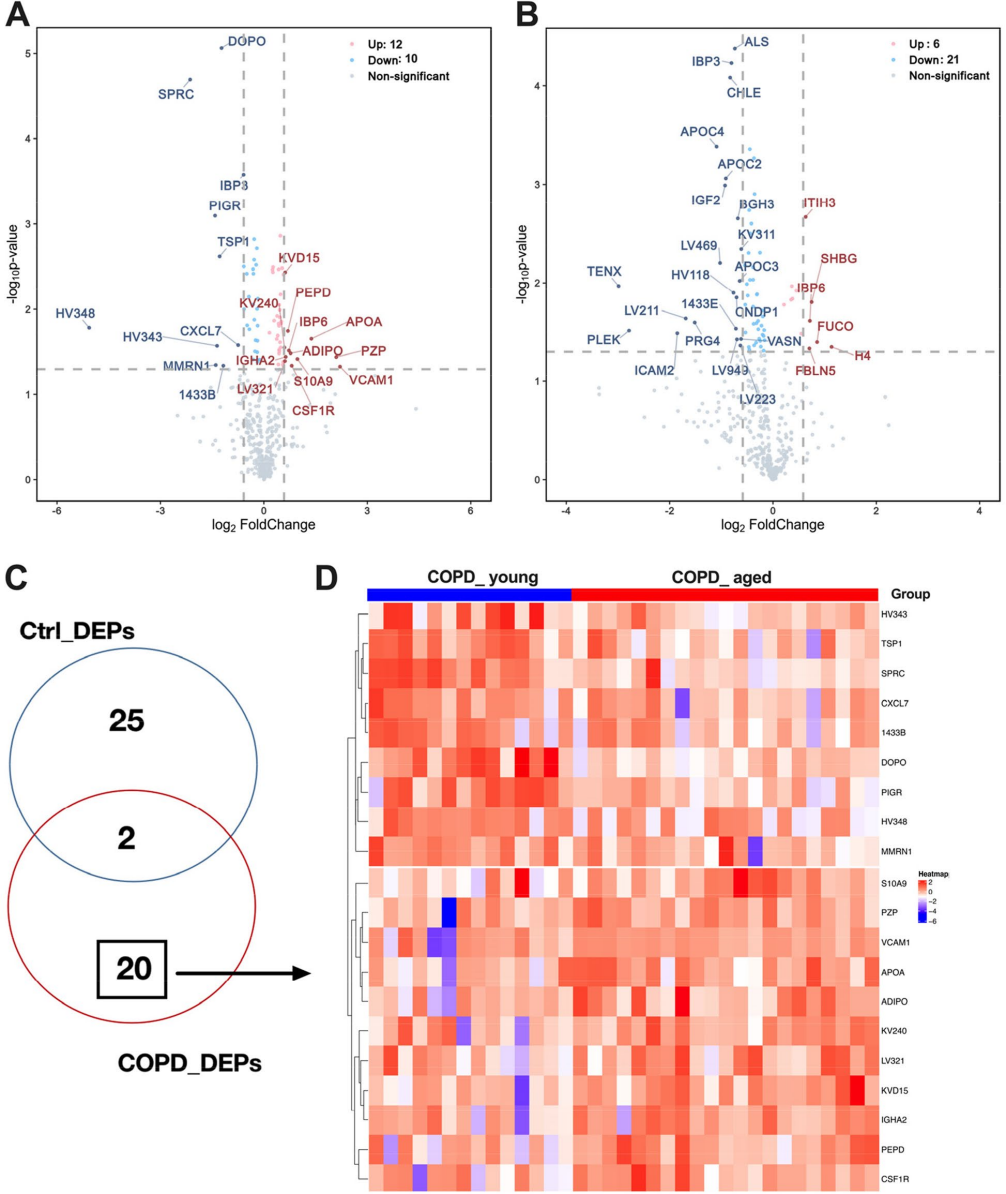
Comparative plasma proteome profiling across experimental cohorts. **A** Volcano plot analysis reveals differentially expressed proteins (DEPs) between COPD-aged and COPD-young groups. **B** Comparative volcano plot demonstrates DEPs in aged versus young control cohorts. **C** Venn diagram identifies 20 aging-associated DEPs specific to COPD progression (intersection of COPD-aged/young and control-aged/young comparisons). **D** Hierarchical clustering of consensus aging-COPD DEPs displays distinct proteomic signatures across cohorts

To delineate aging-specific proteomic perturbations in COPD pathogenesis, comparative plasma proteome analysis was performed across age-stratified cohorts. Volcano plot analysis identified 22 differentially expressed proteins (DEPs) in aging COPD patients (12 upregulated and 10 downregulated) as shown in Fig. 2 A, and 27 DEPs in aging controls (6 upregulated, 21 downregulated; Fig. 2B). Venn diagram intersection revealed 20 conserved aging-associated DEPs in COPD (11 upregulated, 9 downregulated; Fig. 2C), forming distinct expression patterns in hierarchical clustering (Fig. 2D). The detailed information of these 20 aging-associated candidate biomarkers is cataloged in Table 2.

**Table 2:** Detailed information of specific aging-associated DEPs in COPD

PG.ProteinNames	PG.ProteinDescriptions	PG.Genes	log_2_FC	*P*.Value
HV348	Immunoglobulin heavy variable 3–48	*IGHV3-48*	−5.067	0.016
SPRC	SPARC	*SPARC*	−2.138	0.000
PIGR	Polymeric immunoglobulin receptor	*PIGR*	−1.416	0.001
MMRN1	Multimerin-1	*MMRN1*	−1.395	0.045
HV343	Immunoglobulin heavy variable 3–43	*IGHV3-43*	−1.358	0.027
TSP1	Thrombospondin-1	*THBS1*	−1.284	0.002
DOPO	Dopamine beta-hydroxylase	*DBH*	−1.228	0.000
1433B	14–3-3 protein beta/alpha	*YWHAB*	−1.176	0.045
CXCL7	Platelet basic protein	*PPBP*	−0.746	0.026
KV240	Immunoglobulin kappa variable 2–40	*IGKV2-40*	0.595	0.028
LV321	Immunoglobulin lambda variable 3–21	*IGLV3-21*	0.611	0.040
KVD15	Immunoglobulin kappa variable 3D-15	*IGKV3D-15*	0.617	0.004
IGHA2	Immunoglobulin heavy constant alpha 2	*IGHA2*	0.632	0.036
PEPD	Xaa-Pro dipeptidase	*PEPD*	0.697	0.018
ADIPO	Adiponectin	*ADIPOQ*	0.774	0.033
CSF1R	Macrophage colony-stimulating factor 1 receptor	*CSF1R*	0.807	0.045
S10A9	Protein S100-A9	*S100A9*	0.972	0.038
APOA	Apolipoprotein(a)	*LPA*	1.377	0.022
PZP	Pregnancy zone protein	*PZP*	2.098	0.036
VCAM1	Vascular cell adhesion protein 1	*VCAM1*	2.208	0.047

### Functional enrichment analyses

**Fig. 3:**
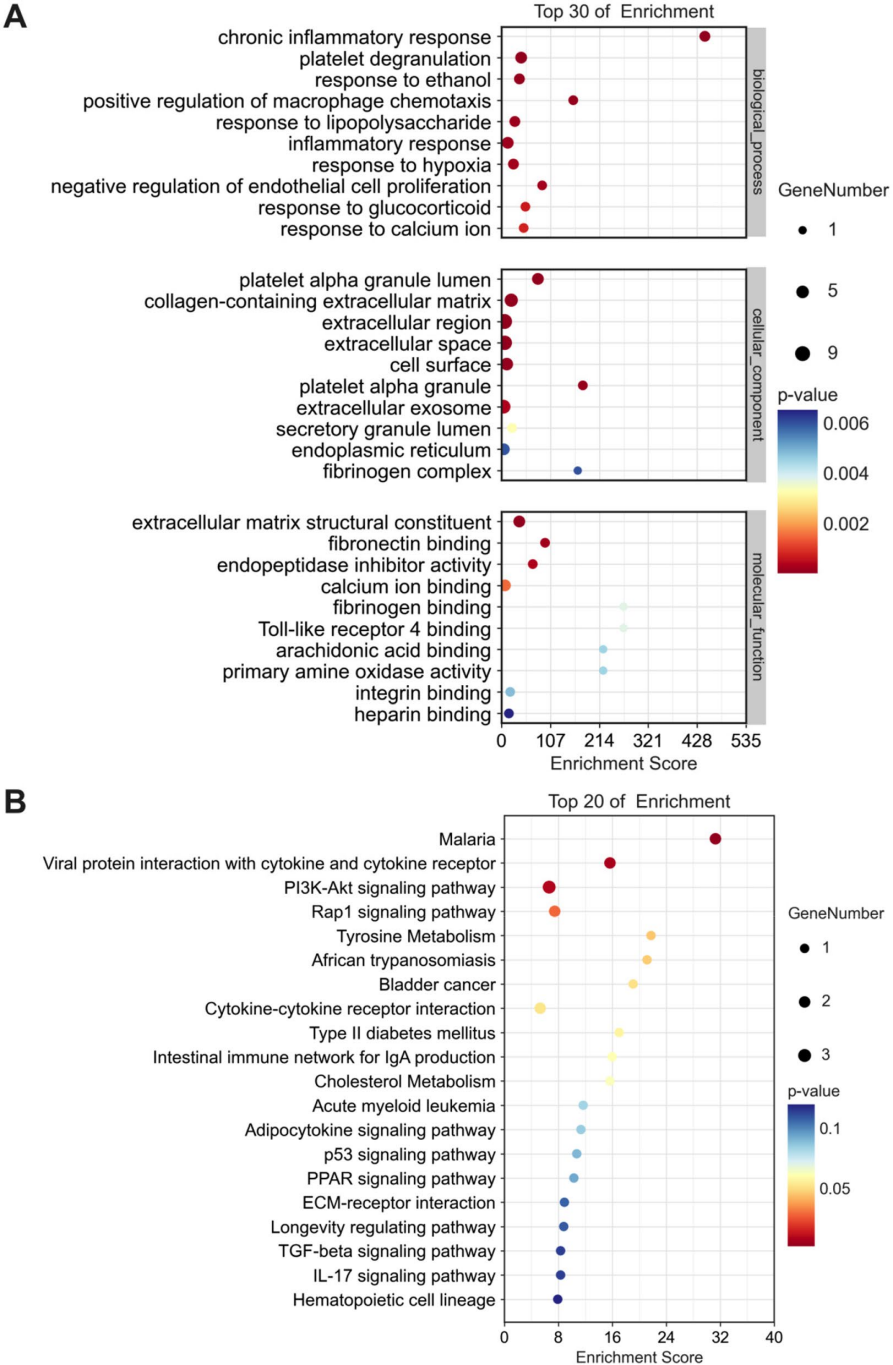
Functional annotation of aging-associated COPD plasma proteome signatures. **A** Topologically ranked Gene Ontology (GO) terms across three domains: biological processes, cellular components, and molecular functions. **B** Kyoto Encyclopedia of Genes and Genomes (KEGG) pathway enrichment analysis reveals 20 significantly dysregulated pathways, sized by gene ratio and colored by enrichment significance

Functional annotation of the 20 aging-associated DEPs revealed distinct pathophysiological signatures in COPD progression. GO enrichment analysis with FDR correction (< 0.05) demonstrated three-tiered functional clustering: In the aspect of biological processes, these DEPs were mainly enriched in chronic inflammatory response, platelet degranulation, regulation of macrophage chemotaxis and endothelial cell proliferation, response to ethanol, hypoxia, glucocorticoid, calcium ion, and lipopolysaccharide. In terms of cellular component, these DEPs were mainly enriched in platelet alpha and secretory granule lumen, collagen-containing extracellular matrix, cell surface, platelet alpha granule, endoplasmic reticulum, fibrinogen complex, extracellular region, space, and exosome. Regarding molecular function, the DEPs were mainly related to extracellular matrix structural constituent, endopeptidase inhibitor and primary amine oxidase activity, fibronectin, calcium ion, Toll-like receptor 4 fibrinogen, arachidonic acid, integrin, and heparin binding (Fig. 3A, Supplementary file 2).

KEGG pathway analysis identified six significantly enriched pathways: Malaria pathogenesis (hsa05144), Viral protein interaction with cytokine and cytokine receptor (hsa04061), PI3K-Akt signaling pathway (hsa04151), Rap1 signaling pathway (hsa04015), Tyrosine metabolism (hsa00350), African trypanosomiasis (hsa05143) (Figs. 3B, Supplementary file 2).

STRING-based protein interaction network analysis revealed a core interactome comprising 9 proteins (Supplementary Figure S1). Notably, TSP1 emerged as topological hubs, suggesting their pivotal role in aging-related COPD pathology.

### Correlation and diagnostic analysis

**Fig. 4:**
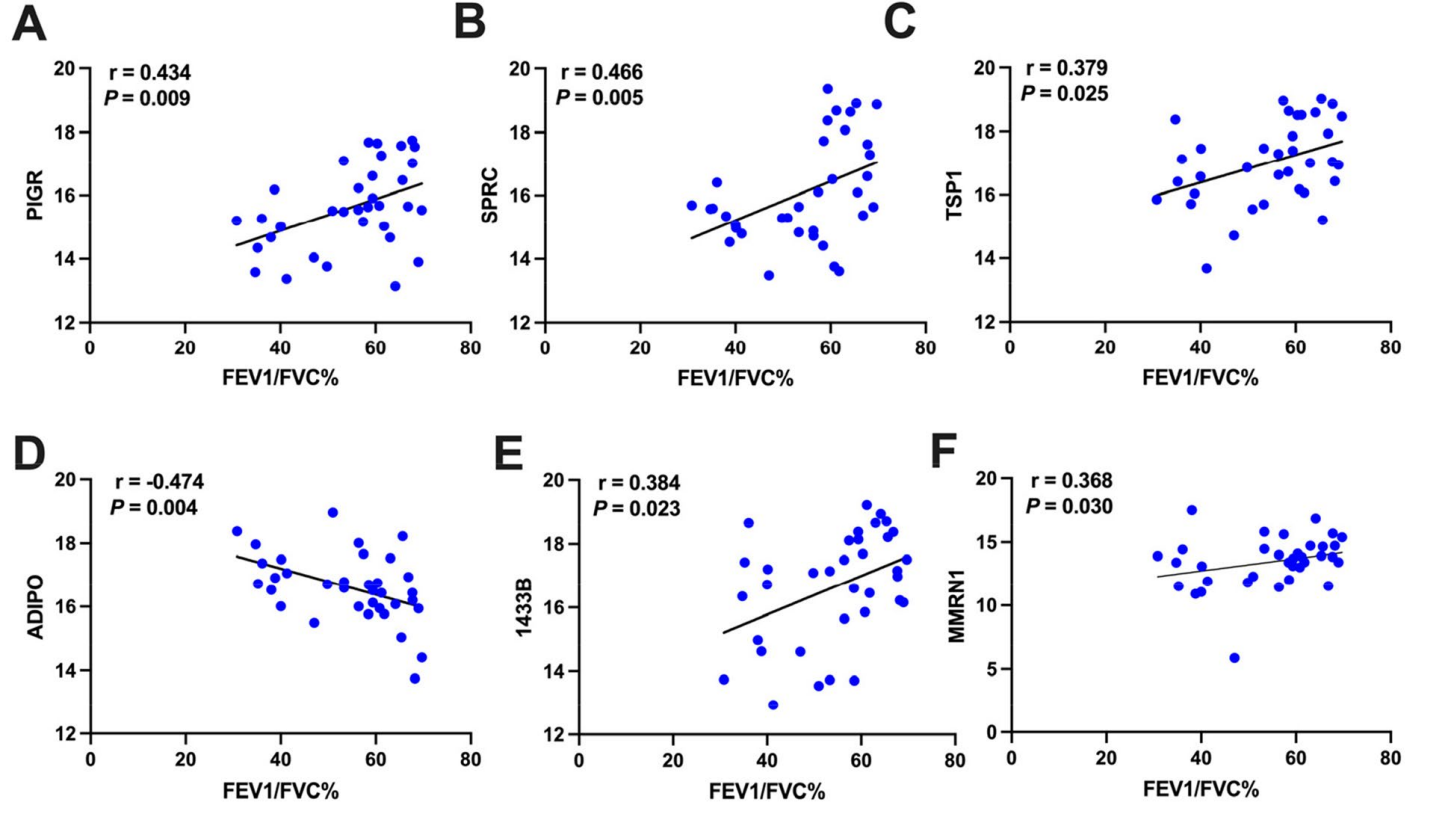
The relationship between the core aging-related DEPs and lung function in COPD patients

Spearman correlation analysis revealed significant correlations between FEV₁/FVC and six of the 20 age-related proteins in COPD patients. Specifically, PIGR, SPRC, TSP1, 1433B, and MMRN1 were positively correlated, whereas ADIPO was negatively correlated (Fig. 4 and Table 3). After adjustment for sex, smoking status, and BMI, multivariable linear regression confirmed that PIGR (β = 2.865, 95% CI: 0.273–5.458, P = 0.031) and ADIPO (β = –4.892, 95% CI: –7.818 to –1.965, P = 0.002) remained independently associated with FEV₁/FVC (Table 4). No significant associations were observed for the remaining proteins after adjustment.

**Table 3:** Spearman correlation analysis between the age-related DEPs and FEV1/FVC ratio in COPD patients

DEPs	Rho	95% Confidence Interval	*P* value
ADIPO	**−0.474**	**−0.702 ~ −0.157**	**0.004**
SPRC	**0.466**	**0.147 ~ 0.697**	**0.005**
PIGR	**0.434**	**0.107 ~ 0.676**	**0.009**
1433B	**0.384**	**0.048 ~ 0.642**	**0.023**
TSP1	**0.379**	**0.042 ~ 0.638**	**0.025**
MMRN1	**0.368**	**0.029 ~ 0.631**	**0.030**
KVD15	−0.322	−0.599 ~ 0.022	0.059
CXCL7	0.312	−0.0345 ~ 0.591	0.069
APOA	−0.281	−0.569 ~ 0.068	0.102
PZP	−0.270	−0.561 ~ 0.0795	0.117
HV343	0.250	−0.101 ~ 0.546	0.148
S10A9	−0.228	−0.529 ~ 0.124	0.187
VCAM1	−0.151	−0.469 ~ 0.202	0.388
DOPO	0.143	−0.209 ~ 0.463	0.412
PEPD	−0.104	−0.431 ~ 0.247	0.550
LV321	−0.095	−0.424 ~ 0.256	0.587
CSF1R	0.078	−0.272 ~ 0.409	0.657
HV348	0.048	−0.299 ~ 0.384	0.785
KV240	−0.026	−0.365 ~ 0.319	0.883
IGHA2	−0.014	−0.355 ~ 0.330	0.936

**Table 4:** Multivariable linear regression nanalyses were performed for the six candidate proteins and FEV1/FVC ratio in COPD patients

Protein Name	β	95% Confidence Interval	*P* value
ADIPO	**−4.892**	**−7.818 ~ −1.965**	**0.002**
SPRC	2.096	−0.081 ~ 4.273	0.059
PIGR	**2.865**	**0.273 ~ 5.458**	**0.031**
1433B	1.896	−0.140 ~ 3.931	0.067
TSP1	2.548	−0.026 ~ 5.194	0.052
MMRN1	0.553	−1.300 ~ 2.405	0.547

**Fig. 5:**
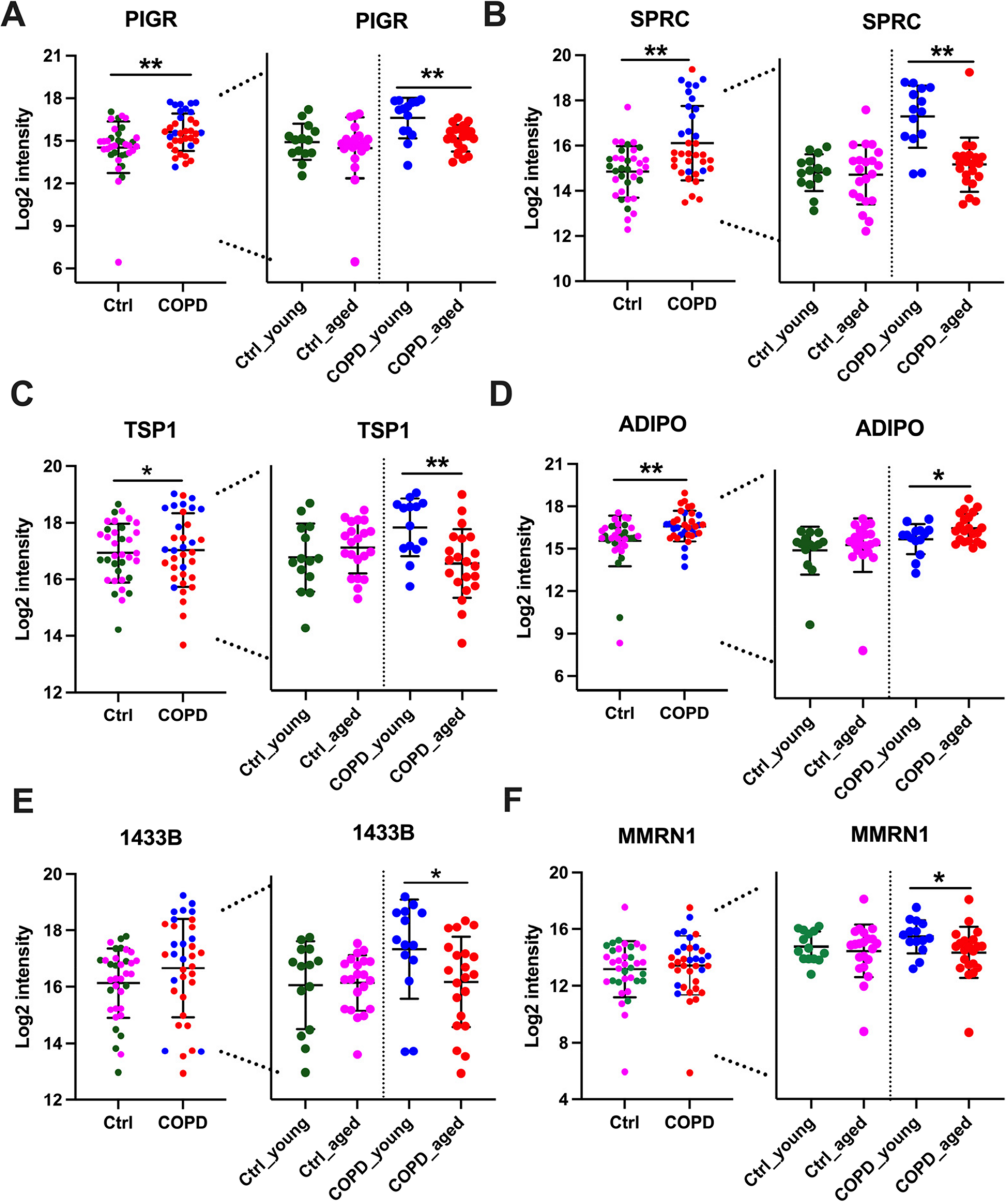
Quantitative profiling of log2-transformed intensities for core aging-related proteins was performed across four age-stratified cohorts. Young controls (Ctrl_young, *n* = 14), aged controls (Ctrl_aged, *n* = 21), young COPD patients (COPD_young, *n* = 14), and aged COPD patients (COPD_aged, *n* = 21). **P < 0.01, *P < *0.05*

To further characterize the expression patterns of these proteins in relation to disease status and age, we next compared their plasma levels across subgroups. Among the above six proteins, the protein intensity dot plots showed that the expression of PIGR, SPRC, TSP1, and ADIPO was significantly upregulated in COPD compared to the control group, as depicted in Fig. 5, whereas those of 1433B and MMRN1 exhibited no significant difference between the two groups. Moreover, patients in the COPD_aged group had significantly lower levels of PIGR, SPRC, and TSP1 than those in the COPD_young group. Meanwhile, a dramatic increase in ADIPO was observed in the COPD_aged group compared with that in the COPD_young group. Collectively, these findings suggest that the pathogenesis of COPD in young patients and aged patients is not precisely the same. Subsequently, we also conducted an analysis at different smoking statuses and GOLD stages within the COPD group. The results indicated that the expression of PIGR, SPRC, TSP1, and ADIPO showed no significant difference in the subgroup analysis (Supplementary Figure S2 and Figure S3). However, there was a decreasing tendency for the expression of PIGR, SPRC, and TSP1, and an increasing tendency for ADIPO expression based on the severity of the disease.

**Fig. 6:**
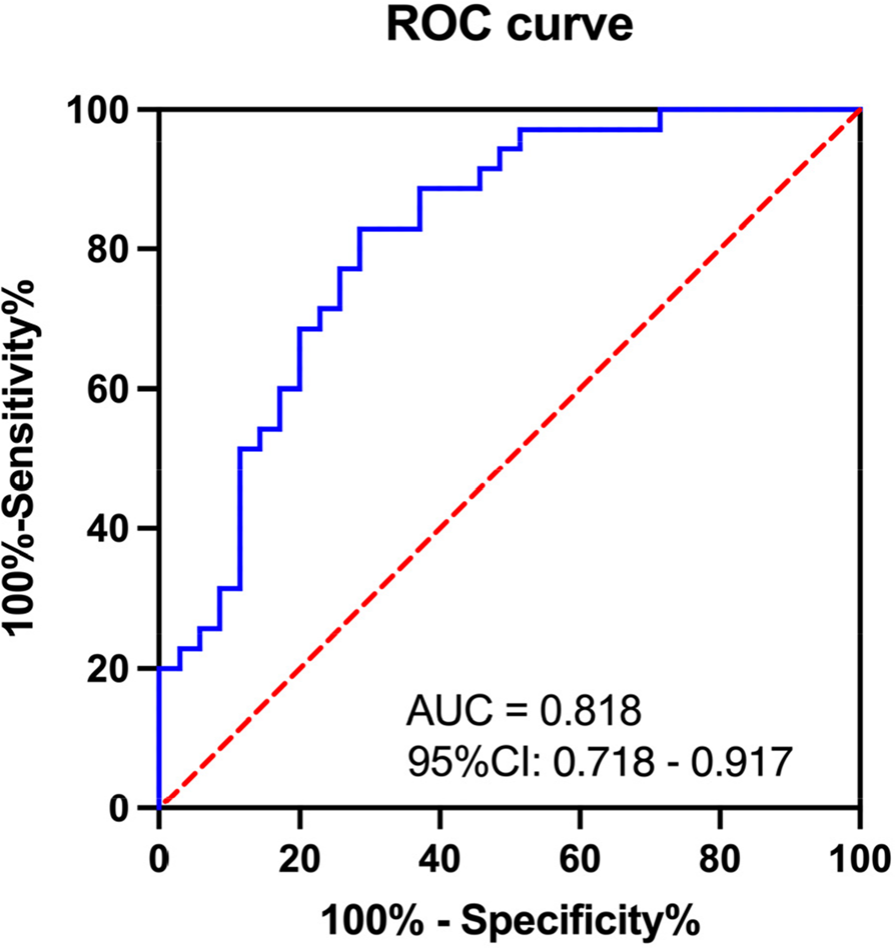
Multivariate predictive modeling integrating critical four age-associated protein biomarkers with age-dependent risk factors for diagnostic analysis of COPD onset

While our primary aim was to identify age-related molecular changes in COPD rather than develop a diagnostic classifier, we explored whether a combination of age and the four proteins showing age-related expression differences (PIGR, SPRC, TSP1, and ADIPO) could distinguish COPD patients from controls. This exploratory analysis was intended to provide a preliminary assessment of their collective discriminatory potential, noting that SPRC and TSP1 showed unadjusted but not adjusted associations with lung function. The model achieved an AUC of 0.818 (95% CI: 0.718–0.917; Fig. 6), suggesting that these age-related proteins capture disease-relevant variation. However, we emphasize that this model requires validation in independent cohorts before any clinical application.

## Discussion

The quality of current COPD care and management systems worldwide remains suboptimal, with over three million annual deaths attributable to this disease [2, 20]. Accumulating evidence indicates that aging not only increases COPD incidence but also complicates its clinical management [9, 10]; however, the mechanistic interplay between aging and COPD pathogenesis remains poorly understood. In the present study, we employed DIA-MS-based plasma proteomic profiling across four age-stratified subgroups to identify 20 age-related differentially expressed proteins (DEPs) in COPD. These DEPs were significantly enriched in pathways associated with inflammatory responses and PI3K-Akt signaling.

Age-related chronic inflammation is well established as a primary driver of various chronic diseases, including COPD [21]. Patients with COPD exhibit elevated systemic and airway inflammation, and persistent inflammatory responses contribute to airway remodeling and small airway obstruction, rendering current therapeutic interventions less effective [22, 23]. Consequently, targeted therapies directed at specific inflammatory pathways represent a promising avenue for future COPD research. In this context, the PI3K/Akt signaling pathway has emerged as an attractive investigational target for inflammatory diseases [24]. Notably, crocin has been shown to exert therapeutic effects in COPD by modulating PI3K/Akt-mediated inflammatory pathways [25], while wortmannin, a phosphoinositide 3-kinase (PI3K) inhibitor, promotes alveolar regeneration and demonstrates potential as a therapeutic approach for COPD [26].

Through stratified subgroup analyses of the plasma proteome, six of the 20 age-related proteins showed significant correlations with FEV₁/FVC in COPD patients and were linked to inflammatory pathways. Among these, four proteins—PIGR, SPRC, TSP1, and ADIPO—exhibited significantly elevated expression in the COPD group compared with controls, whereas 1433B and MMRN1 showed no intergroup differences. Notably, expression levels of PIGR, SPRC, and TSP1 were significantly lower in the COPD_aged group than in the COPD_young group and correlated positively with FEV₁/FVC in univariate analysis. Conversely, ADIPO expression was significantly higher in the COPD_aged group compared with the COPD_young group and correlated negatively with lung function parameters. After adjusting for sex, smoking status, and BMI, PIGR and ADIPO remained independently associated with FEV₁/FVC, whereas SPRC and TSP1 did not reach statistical significance. Importantly, none of these four proteins showed significant expression differences between older and younger individuals in the control group. This distinct expression pattern suggests that the observed alterations are specifically associated with the impact of aging on COPD pathogenesis, rather than reflecting aging per se.

As an exploratory analysis, we constructed a predictive model combining these four proteins with age, given their age-related expression differences and biological relevance to COPD pathophysiology. This model demonstrated reasonable discriminatory ability (AUC 0.818). This proof-of-concept finding suggests that integrating multiple age-related proteins may capture aspects of COPD pathophysiology more effectively than individual proteins alone. However, we interpret these results cautiously, emphasizing the need for validation in larger, independent cohorts before any clinical application can be considered.

The four proteins identified are established rather than novel biomarkers in COPD pathogenesis, with accumulating evidence consistently implicating their altered expression in disease progression. For PIGR, previous studies have reported increased levels in lung tissues, sputum, and plasma of smokers and smokers with COPD compared to nonsmokers [27], as well as associations with frequent exacerbations [28]. Conversely, Pilette et al. [29] reported reduced PIGR levels in the airway epithelium of COPD patients, potentially impairing mucosal immunity and increasing susceptibility to respiratory infections. Furthermore, pIgR/SIgA deficiency in the airways drives persistent innate immune activation against resident lung microbiota, contributing to progressive small airway remodeling and emphysema [30, 31]. Supporting this, pIgR(-/-) mice, which lack SIgA, spontaneously develop COPD-like pathology with aging. In our study, plasma PIGR expression was significantly elevated in COPD patients compared to controls but was reduced in aged COPD patients relative to their young counterparts. SPRC (also known as SPARC) is an extracellular matrix glycoprotein involved in cell adhesion, tissue remodeling, regulation of cell proliferation, and immune/inflammatory response activation [32, 33]. Impaired core-fucosylation of SPARC has been linked to reduced collagen binding and alveolar structural changes in COPD [34]. TSP1, another extracellular matrix glycoprotein, participates in cell adhesion, angiogenesis, and inflammation [35], with increased expression observed in the lungs of COPD patients [36]. ADIPO, recognized for its anti-inflammatory effects, has emerged as an attractive biomarker for metabolic disorders in COPD [37, 38]. Studies have demonstrated significantly increased serum adiponectin levels in COPD patients, with concentrations correlating directly with disease severity [39] and accelerated lung function decline [40]. Consistent with these findings, we observed elevated plasma levels of all four proteins in COPD patients compared to controls, with ADIPO showing further elevation in aged versus young COPD patients. However, the mechanisms underlying the reduced PIGR, SPARC, and TSP1 concentrations specifically in elderly COPD patients remain unclear and warrant further investigation.

It is important to acknowledge that our study design stratified participants based on chronological age (≤ 50 years vs. ≥ 70 years), which does not necessarily equate to biological age. Biological aging is a heterogeneous process, and individuals of the same chronological age may exhibit different degrees of biological aging, particularly in the context of chronic diseases like COPD where premature aging may occur [6, 9]. Therefore, while our approach successfully identified proteins whose expression patterns differ between young and aged COPD patients, these should be interpreted as age-related changes associated with the disease rather than definitive markers of biological aging per se. Future studies incorporating validated measures of biological age, such as epigenetic clocks or telomere length measurements, would be valuable to determine whether these proteins correlate more strongly with biological age than chronological age. Additionally, incorporate multivariable adjustment for independent validation and unsupervised clustering analyses could potentially reveal subgroups based on proteomic profiles that may better reflect biological aging status, and we plan to explore this approach in future larger cohort studies.

## Conclusions

In this study, a plasma proteomics approach was employed to identify age-related protein biomarkers in COPD. Our findings reveal distinct protein expression profiles between young and aged individuals with the disease. Four proteins—PIGR, SPRC, TSP1, and ADIPO—showed age-dependent expression differences and were associated with lung function, with PIGR and ADIPO demonstrating independent associations after covariate adjustment. Collectively, these proteins represent candidate age-related biomarkers in COPD. Rather than serving as simple diagnostic tools, they may hold potential for patient stratification and endotyping, particularly in older populations, although further validation in independent cohorts is warranted.

## Supplementary Information

Supplementary Material 1.

Supplementary Material 2.

Supplementary Material 3.

## Data Availability

The datasets generated and/or analysed during the current study are available in the iProX partner repository (accession: PXD070723).

## Abbreviations

COPD: Chronic obstructive pulmonary disease
FEV1: Forced expiratory volume in 1st second
FVC: Forced vital capacity
DEPs: Differentially expressed proteins
FC: Fold change
GO: Gene ontology
KEGG: Kyoto Encyclopedia of Genes and Genomes
ROC: Receiver Operating Characteristic
AUC: Area Under the Curve
KNN: K-nearest-neighbor
DIA: Data-Independent Acquisition
LC MS/MS: Liquid Chromatography Tandem Mass Spectrometry Analysis
EDTA: Ethylenediaminetetraacetic acid
PPI: Protein-Protein Interaction Networks
