# Supplementary material for: Plasma proteomic profiling uncovers the age-related molecular changes in chronic obstructive pulmonary disease
Source: BMC Pulm Med. 2026 Jun 10;26:354. doi: 10.1186/s12890-026-04396-8 (PMC13479710; doi:10.1186/s12890-026-04396-8)
Supplement: Supplementary file 1 [file 12890_2026_4396_MOESM1_ESM.docx]

**Supplementary figures**

**Figure S1. Protein-protein interaction (PPl) network analysis of aging-related DEPs in COPD via String online database.**

**Figure S2. The expression of core aging-related proteins analyzed at different status of smoking in COPD patients.** COPD patients (n=35), No smoking (n=20), Smoking (n=15). ***P* <0.01*, *P* <0.05*.*

**Figure S3. The expression of core aging-related proteins analyzed at different GOLD stages of COPD**. COPD patients (n=35), GOLD 1 (n=5), GOLD 2 (n=15), GOLD 3 (n=10), GOLD 4 (n=5). ***P* <0.01*, *P* <0.05*.*
